# A new starch: Dynamics of Arabidopsis starch metabolism are influenced by the oligosaccharide pool

**DOI:** 10.1093/plphys/kiaf085

**Published:** 2025-03-05

**Authors:** Jiawen Chen

**Affiliations:** Assistant Features Editor, Plant Physiology, American Society of Plant Biologists; Division of Crop Biotechnics, Department of Biosystems, KU Leuven, 3001 Leuven, Belgium

Plants use starch as a stable form of carbohydrate storage to regulate energy balance and support plant growth when photosynthesis is not available. Typically starchy tissues such as wheat grains, maize kernels, and potato tubers are abundant in storage starch, which is stored for long periods of time until it is used for germination and sprouting. Most green plants also have starch in the leaf chloroplasts, where starch is used as an energy source during the night. Starch consists of 2 types of glucose polymers: linear amylose connected by α-1,4-glycosydic linkages, and branched amylopectin, which also has α-1,6-linked branches. These polymers are arranged in a semi-crystalline structure and assembled into insoluble granules.

Arabidopsis starch biosynthesis and degradation is a tightly regulated process, with plants adjusting their rate of nighttime starch degradation according to the length of the night (Graf et al. 2010). Starchless Arabidopsis mutants have defective growth phenotypes under short days (Caspar et al. 1985), as they do not have enough energy to sustain the long night. Although wild-type Arabidopsis generally has a net synthesis of starch during the day and net degradation of starch during the night, we do not yet fully understand the dynamics of these processes. Starch synthesis and degradation enzymes are both present in the chloroplast throughout day/night cycles, and there is evidence for some starch degradation during the day (Fernandez et al. 2017). At the end of the night, Arabidopsis leaf starch is not fully depleted. Therefore, there seems to be a constant growth and shrinkage of starch granules throughout diurnal cycles (Burgy et al. 2021).

Starch initiation determines granule number, size, and shape. There are several proteins required for this process, and mutants missing any of these have a reduced number of granules per chloroplast (Seung and Smith 2019). The priming of starch granules could happen de novo through a yet unknown process or through maltooligosaccharides (MOS) generated from starch degradation products. Starch degradation is a stepwise process involving different enzymes that help release glucan chains or break them down into smaller units. Isoamylase 3 (ISA3) and limit dextrinase (LDA) are debranching enzymes that cleave α-1,6-linkages. α-Amylase 3 (AMY3) cleaves internal α-1,4-linkages and releases a mix of linear and branched MOS. In *isa3lda* mutants starch degradation is impaired, leading to starch accumulation. Branched MOS are particularly increased at the end of the night in these mutants (Delatte et al. 2006). These MOS are released from the granule surface by AMY3. In *isa3ldaamy3* triple mutants starch degradation is even more impaired than *isa3lda* double mutants. However, *amy3* single mutants do not have a starch degradation phenotype (Streb et al. 2012).

**Figure 1.**
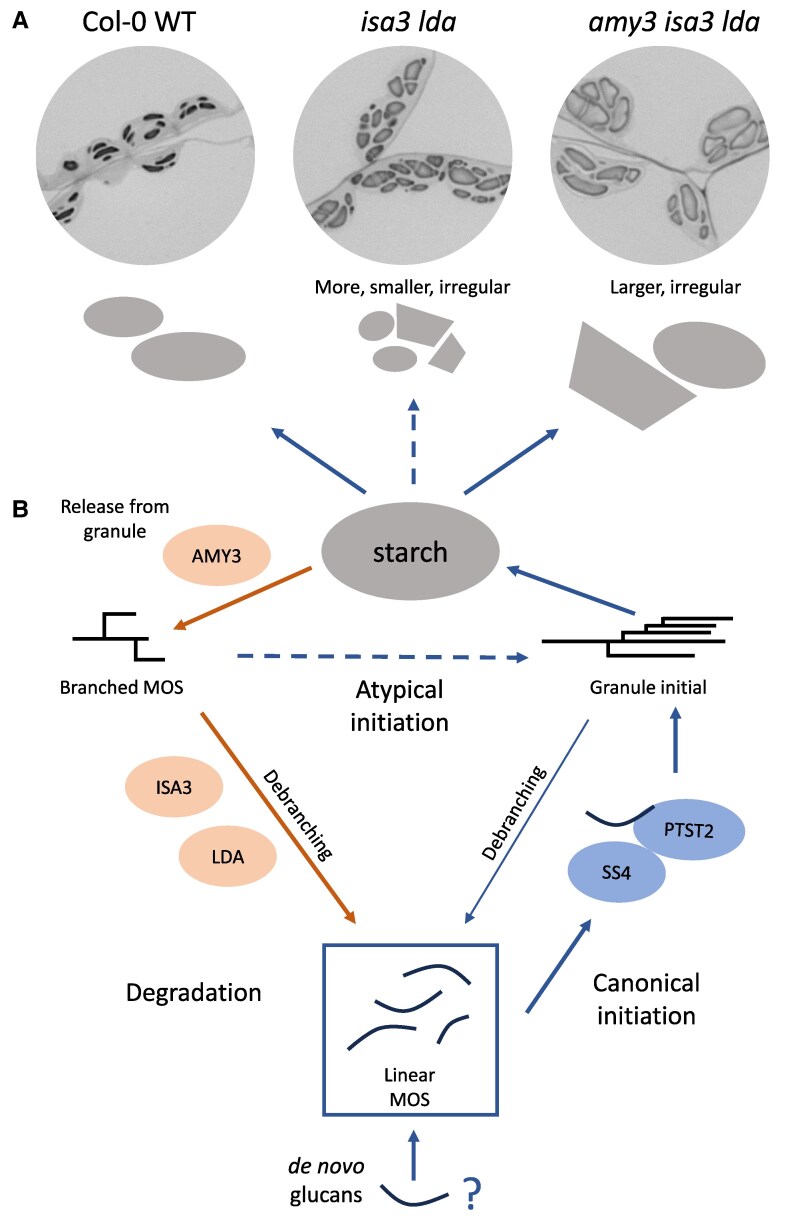
**A)** Diagrams and light microscopy images of WT and mutant Arabidopsis leaf starch granules at the end of the light period, starch stained with toluidine blue, reproduced from Heutinck et al. (2024). **B)** Schematic of granule initiation and degradation pathways, starting from a pool of MOS that can come from de novo primers or as products of starch initiation or degradation pathways. Blue arrows = initiation, orange arrows = degradation. Dashed arrows = atypical initiation.

In this issue of *Plant Physiology*, Heutinck et al. further characterized the Arabidopsis *isa3lda* and *isa3ldaamy3* mutants, looking at their leaf starch granule size distribution using flow cytometry (Thieme et al. 2023) and studying granule morphology using light microscopy, transmission electron microscopy, scanning electron microscopy, and serial block face scanning electron microscopy. They saw that *isa3lda* mutants not only had increased branched MOS and overall starch content, but the mutants also had an increased number of starch granules compared with the wild type (WT), especially small granules (Fig. 1A). The increased granule number suggested that these mutants affected granule initiation events as well as degradation. The increased granule initiation could be caused by an increase in the total pool of soluble MOS, resulting in unregulated priming events. The *isa3lda* granule shapes were also irregular, with some granules that looked like the lenticular granules in WT and others that were more polyhedral in shape (Fig. 1A).

The authors found that the irregular initiations in *isa3lda* were formed by a separate pathway to the canonical granule initiation pathway (Fig. 1B). Granule initiation in WT requires the enzyme Starch Synthase 4 (SS4), and glucan substrates are brought to SS4 by the protein PTST2 (Seung and Smith 2019). Mutants in either SS4 or PTST2 have fewer, larger granules than WT. The authors generated *ss4isa3lda* and *ptst2isa3lda* mutants, and both mutants still had a similar high number of granules as *isa3lda*. Therefore, the extra initiations in *isa3lda* were not facilitated by SS4 and PTST2 but by an alternative, unregulated initiation pathway. These irregular initiations could be caused by other starch synthesis enzymes. Interestingly, *ss4* mutants have round rather than lenticular WT granules, and this round phenotype persisted in *ss4isa3lda*. Although the alternative granule initiation pathway could prime new granules without SS4, it could not compensate for the role of SS4 in controlling lenticular granule shape (Burgy et al. 2021). The alternative granule initiation also resulted in uncontrolled localization of the granules. WT granules are typically formed in pockets between thylakoid membranes, but in *isa3lda* some of the granules initiated adjacent to the chloroplast envelope membrane.

The *isa3lda* mutants could not form as many alternative granule initiations if they also lacked AMY3. In *isa3ldaamy3* triple mutants there were fewer granules than in *isa3lda*, as the branched MOS were no longer released from the granule by AMY3 (Fig. 1). The number of granules in *isa3ldamy3* was only slightly higher than in WT, but the granules were much larger compared with both WT and *isa3lda*. Therefore the previously observed high starch content in *isa3ldaamy3* mainly resulted in an increase in granule size. The granule shapes were still irregular in *isa3ldaamy3*, similar to *isa3lda*. These results suggest that AMY3 has a unique role depending on the context. AMY3 impairs starch initiation in the absence of SS4, as *amy3ss4* mutants have more granules than *ss4* (Seung et al. 2016). In contrast, the current study suggests AMY3 could promote an SS4-independent initiation pathway by influencing the pool of MOS.

This paper sheds light on the complicated dynamics of MOS metabolism in connection to starch initiation and degradation. The authors observed that the starch degradation mutant *isa3lda* accumulated branched MOS that serve as primers for an unregulated, alternative starch granule initiation pathway, independent from the canonical SS4-PTST2–dependent pathway. Both branched and linear MOS can contribute to the total MOS pool that is available as substrates for starch synthesis enzymes (Fig. 1). These MOS can come from glucan products generated during granule initiation and degradation. Any process that influences the MOS pool may have an impact on granule initiation. Future studies will need to determine what roles different MOS species play and which proteins are responsible for the alternative granule initiation. These could include starch synthases and branching enzymes. As we learn more about starch metabolism, we can build an increasingly dynamic picture of this process.
